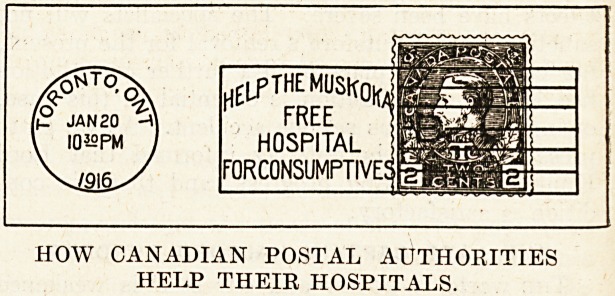# Hospital and Institutional News

**Published:** 1916-03-11

**Authors:** 


### HOSPITAL AND INSTITUTIONAL NEWS.

### LORD KNUTSFORD.

We are indeed sorry to learn that the concussion from which Lord Knutsford is suffering and its effects have been severe. The specialists will not sanction Lord Knutsford's removal for the present, nor can this take place until a further consultation has been held. Caution is essential in this case, owing to a previous serious accident. As we go to press we are authoritatively informed that Lord Knutsford is making progress, and that his condition is satisfactory.

### THE LATE CAPTAIN HALFORD BURDETT.

The world is poorer and our staff is weakened by the startlingly sudden and unexpected death of this gallant officer, who died on the 3rd inst. in the service of his country. Expressions of regret have reached us from the Master of Pembroke College, Cambridge, where he spent happy years and had many friends, and Mrs. Hadley, solicitors testifying to the value of his co-operation in important cases, his colleagues and fellow-workers at the Board of Education, and also very many others from those who knew him in all ranks of society from the highest to the lowest. Captain Burdett's personality possessed a special charm, and made him beloved by all who were fortunate enough to have been associated with him at college, at the Bar, in the Army, in business, as a sportsman, in society, or as a friend ever ready to do a good turn to all and sundry. He was an able speaker, a clear and forcible writer, and we shall find it hard, if not impossible, to fill his place on the staff. He was only thirty-eight, and had just made arrangements to his satisfaction and pleasure, whereby his promotion and future work after the war were secured. He was buried at Rusthall, near Tunbridge Wells, on the 6th inst.; and the attendance at his funeral revealed the widespread sorrow and regret caused by his death amongst his comrades-in-arms and all classes resident in and around the district. He has left a widow and three sons full of promise, one of whom is preparing to enter the Navy, and the other two, though very young, are keen on joining the Army. On behalf of our staff and ourselves we offer the sincerest sympathy and condolences to Mrs. Halford Burdett and her children.

### RESEARCH IN MILITARY SURGERY.

With the co-operation of the Medical Research Committee set up originally in connection with the working of the National Health Insurance Act, a system has lately been adopted by which research into selected diseases or injuries occurring in soldiers at the seat of war will be much facilitated. Attention having been directed to some particular kind of wound, for instance, care is taken that all such cases shall be very carefully annotated in base hospital or casualty clearing station. Then on transfer of the individual to England he is provided with a stamped postcard addressed to the Medical Research Committee, which the medical officer into whose charge he comes is expected to fill in and post. This card merely informs the authorities of the destination at which the patient has arrived. When it has been duly sent off, in a few days the medical officer receives a form with spaces provided for the answering of many questions about the patient's condition. These questions are not expected to be answered until the patient's convalescence is far advanced, as many of them refer to his progress under treatment and to the methods of treatment themselves. The scheme is carefully thought out, and given loyal co-operation by the medical officers should have valuable results.

### WHAT IS A "NATIONAL NECESSITY"?

Many strange statements have been made during the last two or three weeks before the local tribunals which have been set up in connection with the National Service Act. A patent-medicine manufacturer who appealed recently for the exemption of his son claimed that his product is a "national necessity." There is no need to suppose that this declaration was not sincerely made, for patent-medicine manufacturers ascribe such astonishing virtues to their wares in their advertisements that they may well come in time to believe every word of them as true. There are, however, unfeeling persons who denounce all patent medicines in bulk as unnecessary and potentially harmful; and even short of such an uncompromising attitude it is difficult to maintain that a particular one of the hundreds of mild aperient preparations on the market is in any proper sense of the words a "national necessity." Without any disrespect to the sincerity of the appellant in this particular case, we feel that the tribunal dealt generously with him in granting a two-months' postponement, and that the need for men is a much more vital necessity to the nation at the moment than even the manufacture of his doubtless excellent specific.

### A "CERTAIN LIVELINESS" AT CARDIFF.

Turning over the pages of the latest report issued by the board of management of King Edward VII.'s Hospital, Cardiff, we are struck by the liveliness with which new developments of the work are being carried out. In small things as in great, a central driving force radiates energy in a widening circle. A few instances of this invite reproduction and study. By the institution of what is known as the "Hospital Penny," the Cardiff and District Hospital Society has developed a new source of income which, from the two collieries which participate in the scheme, has amounted to £400, with the result that other collieries are considering its adoption also. Again, through an anonymous gift of £5,000, the Maternity Flat, a scheme cherished since 1911, when the Marchioness of Bute was promised that it should be established, is now in being, thus paving the way for the extension scheme which The Hospital has previously described. A small but desirable improvement has also occurred through the endowment fund lately established for the provision of a gold and a silver medal to be awarded to the best nurses of each year; while the gift of "Bedford House," which now provides ten beds for wounded officers, will enable the hospital after the war to add to its departments a home for paying patients, an experiment which the board are contemplating in accordance with the desire of the donor, Mr. Leyton Thomas. Another point must also be mentioned. In dealing on February 26 with the recent opening of the new wing it was remarked that the only enemy of the Welsh National School of Medicine was the Government. The efforts on every side which the hospital has made to conciliate the Treasury are well illustrated by the assurance which has been given that, if only permission be granted to complete the building of the school, no man eligible for military service will be employed in the building operations. This is a straw which shows which way the wind of enthusiasm is blowing, and the measure of the recent disappointment may be guessed by a little fact of this kind.

### ADVERTISING FOR A FARM.

Most hospitals nowadays have convalescent homes attached to them, but those which do not may be interested in the plan adopted by the committee of the Coventry and Warwickshire Hospital, which is to send their convalescents to recruit at a farm with which a regular arrangement is made. The difficulties attending such a plan are those which result from all temporary arrangements, and, as an instance of this, the tenant of the farm to which the Coventry Hospital's convalescent patients are sent is giving up possession on Lady Day. The hospital authorities, therefore, are advertising for another suitable farm where similar arrangements may be made. In these days, when new or additional sources of income are being desired by nearly everyone, there should be more than one farmer who would be glad to come to terms and devote his surplus accommodation, if suitable, to taking convalescent patients. Such a plan, being properly organised on a regular basis, should be more attractive than the usual haphazard letting of rooms by farmers in the summer. The patients, too, must often prefer the natural amusement provided by farm activities to the organised games which replace them in regular convalescent homes.

### A SECRETARY'S "SALARY" OF £15 A YEAR.

After twenty years' work in the double capacity of honorary secretary and treasurer, Mr. Lanfear R. Tanner has retired. During this period the Frome Victoria Hospital has been moved from its original premises in Castle Street to its present site, where an extension scheme has only lately been completed. An honorary secretary is naturally a part-time secretary as a rule; and, except with paid assistance, can hardly undertake the work of a growing hospital. In appointing a successor the committee of the hospital have selected a lady, Mrs. Porter, at a commencing salary of £15 a year. Such a sum may be termed an honorarium; it cannot be described as a salary, for work requiring a trained secretary cannot be performed for the equivalent of a probationer's pocket-money.

### AN ABSOLUTELY ORIGINAL APPEAL.

### HOW CANADIAN POSTAL AUTHORITIES HELP THEIR HOSPITALS.

The war has knit together the Empire, and made the inhabitants of all the Dominions and islands which constitute it one people. This reuniting of the mother and her children, this quickening of the hearts of the race and peoples, has already produced striking results, which will multiply and grow after the war. The children nowadays tend more and more to become the rulers of the household. They not unnaturally and necessarily have exhibited an originality of resource and a quickened energy which not a few times in the past has made them leaders of the Old Country. Where else but in one of the Dominions beyond the seas should we find the Post Office consenting to help the finances of a Free Hospital for Consumptives by placing on sale a postage stamp at a price which franks the letter and leaves something for the local hospital? The children of the Empire have moved their rulers to sanction this form of appeal, which has the twofold advantage of making it easy for every resident to hasten the opening and provide the maintenance necessary, whilst leaving the revenue raised through the Post Office at least as great as, or probably greater than, that which would otherwise be derived from the sale of ordinary postage stamps in the ordinary way. This novel method of protecting a community by providing for the care of the tubercular through the Post Office is remarkable for its ingenuity, which takes away from the postal authorities the obstructive policy they have pursued invariably towards efforts of this kind. It effectively secures this triumph by removing all the objections to the plan which those authorities have enumerated in defence of their refusal to allow the machinery of the Post Office to be used for any other purpose but the raising of revenue for the ruling authorities of the State.

### THE GROWTH OF ORAL SURGERY.

Mr. L. S. Cohen, chairman of the committee of the Dental Hospital, Pembroke Place, Liverpool, has announced their intention of establishing a department of oral surgery at the hospital for the treatment, by surgeon and dentist, of injuries to the jaws and face of soldiers returned from the front. In supporting the proposal, the Lord Mayor, Alderman Mather, stated that certain facial injuries yielded only to the combined treatment of dentist and surgeon, and that many military surgeons were of opinion that every battalion should have a dental surgeon attached to it. In an interesting speech on the same occasion, Mr. Cohen referred to a visit paid by Mr. Woods and Mr. Gilmour to the base hospitals in France, as a result of which, with the support of the staff, they hoped to establish a department at the hospital to treat serious injuries to the face and jaw when these patients arrived in England. He alluded also to the photographs which had been taken and the models which had been made to show the degree of success with which such injuries could be repaired. Mr. W. H. Gilmour, director of dental education, also urged the need for this department of oral surgery, and pointed out that the proportion of facial and head injuries was much greater in this than in previous wars. An appeal is to be issued.

### A HOSPITAL CHAIRMAN'S UNIQUE EXAMPLE.

Medical officers rarely become chairmen; indeed, between the medical and the administrative staffs of a hospital a gulf is often observed; and the last man to have the smallest inside knowledge of hospital management is very often a doctor "on the staff." It is pleasant, therefore, to record an illustration—may we not rather say an example?—all the other way. The late Sir G. A. Pilkington, who died on January 28, was not only a medical man, but a hospital chairman of very considerable experience. His services, in fact, to the Southport Infirmary were so various that a record has been preserved of the numerous offices he held. It is probably unique, as our readers will judge after reading it:—From 1870-5 he was house surgeon; 1875-9, out-visiting physician; 1880-5, honorary medical officer; 1884, president as mayor; 1886-1916, honorary consulting medical officer; 1892, president as mayor (when he laid the foundation-stone of the new infirmary); 1893-1916, trustee; 1901-1916, chairman of the board of management. This is, for a medical man, surely a unique record. We wish it were not so; but the late Sir G. A. Pilkington's name will always be remembered among hospital chairmen for the example he has set.

### A CHAIRMAN ON THE SPOT.

The good hospital officer, no matter what his position or how occupied, is always thinking of his institution. For instance, at the annual meeting of the Ancoats Hospital, Manchester, Sir Frederick Cawley, Bart., M.P., who presided, told the following incident. Having journeyed direct from London for the meeting and being fifteen minutes late, he apologised by saying that though the railway company and the weather were to blame, the delay had not been altogether fruitless. While waiting at Birmingham for the train to proceed he had informed a gentleman where he was going, whereupon the gentleman kindly promised him that he would forward a donation of £5 to the hospital that day. As an example of being on the spot this could hardly be beaten.

### THE EXTENSION AT ANCOATS HOSPITAL.

While on the subject of Ancoats Hospital allusion should be made to the extension of the out-patient department. The extensions, which should add materially to the effective working of the hospital, consist in the erection of two examination rooms for surgical and medical cases, measuring respectively 20 feet by 15 feet and 18 feet by 15 feet; a large and well-equipped pathological laboratory, and a room in which is installed the cardiographic apparatus recently presented to the institution. There is also a "dark room" provided for "photographic" and special throat examination purposes. All these rooms, approached by means of a wide corridor leading from the patients' waiting hall, are well lighted from the side and have large ventilated lantern lights in addition. The five smaller examination rooms have been remodelled, the roofs reconstructed, and lantern lights inserted. The whole of the new work has been carried out in a similar manner to the older portion of the building, the wall surfaces throughout being finished in glazed tiles from floor to ceiling. The architect is Mr. W. Cecil Hardisty, F.R.I.B.A., of Manchester, who also designed and carried out the erection of the out-patients' block in 1898.

### "DISCHARGED FOR DRUNKENNESS."

Certain points of interest are prominent in the latest report of the Devonshire Hospital and Buxton Bath Charity. The new mineral-water baths have been employed since Whitsuntide last year, and the more seriously crippled patients are mentioned as deriving peculiar benefit from the treatment. More than 1,000 soldiers suffering from rheumatism and allied diseases have been admitted since the war began. It is regrettable to learn that nine patients had to be discharged for drunkenness—a condition, we had hoped, into which no hospital patient could nowadays have any opportunity to fall. In the medico-electrical department, radiant-heat baths and electrical massage have been given, and the equipment includes mechanical vibrators, galvanic and faradic switch-boards, high-frequency apparatus, arthromotor, and electrical cautery.

### THE NECESSARY COMPLEMENT TO SCHOOLS FOR MOTHERS.

The Manchester City Council is considering a proposal made by the Sanitary Committee to increase by £1,500 the grants made to the Schools for Mothers, so as to provide for the retention of eighteen beds at the Babies' Hospital. Dr. J. Niven, the medical officer of health, has put the case so well that we cannot do better than summarise his report on the subject. At present no other express provision is made in Manchester for the treatment and study of babies—that is, children under two years old. Suffering as they do from the various effects of malnutrition, the other hospitals cannot find room for all these cases. At present twelve cots are occupied, and the cases are largely sent to the School for Mothers, which finds the Babies' Hospital a necessary complement to its work. The Local Government Board are prepared to give a grant under their maternity and child-welfare scheme; but, as Dr. Niven reminds the City Council, such grants are only given to sanitary authorities which secure beds and pay half the cost of maintaining them. That is why he is anxious that eighteen beds should be retained by the Corporation. The directors will pay half the cost per bed, and the Corporation would pay the remaining half if the Local Government Board refunded half the amount paid. The net cost to the Corporation would be £350; and, though the Finance Committee recently rejected the proposal, the Corporation has decided to adopt it. A sufficiently large and properly equipped Babies' Hospital is found to be the necessary complement of a School for Mothers in much the same way that school treatment was found a necessary complement to school inspection.

### THE CENTRE OF ATTRACTION IN HOSPITAL WORK.

Good things and evil are often found to come in crowds: it never rains but it pours, as the saying is. A happy instance of the pleasant side of this truth is found at Wolverhampton, where the Midland Counties Eye Infirmary has hardly had time to congratulate itself on a successful year, in which practically every source of income has shown an increase, before news comes that the hospital is about to receive what Mr. A. B. Bantock has described as "the largest legacy which has ever been left to it." The amount of the legacy has not been disclosed, but the hospital's supporters should not fail to draw the moral, which is this: The best way to attract subscriptions and legacies is to invite them by a vigorous and enthusiastic policy of work, for there is as truly a centre of attraction in the matter of subscriptions as there is a centre of gravity in the earth itself.

### KING'S COLLEGE HOSPITAL.

At the annual meeting of King's College Hospital, held on March 2, a somewhat unsatisfactory financial situation was disclosed. The report stated that there has been no diminution of the debt with which the committee were faced at the beginning of 1915; indeed, it has increased, for the excess of expenditure over income was more than £8,000. This deficit is attributed mainly to a falling-off in subscriptions and donations: legacies, too, turned out poorly, for they brought in but £2,578. The Rev. A. C. Headlam, who presided, described the financial position of the hospital as precarious. In spite of the greatly enhanced cost of drugs, provisions, and other necessaries, the hospital was actually run at a lower cost per patient in 1915 than in any year since 1910. The courage of Mr. Headlam in facing an unpalatable situation and stating it in plain terms is highly commendable. We trust that his outspokenness may do good by helping people to realise the true situation of this (and of other) hospitals, and have the effect of loosening purse-strings for the benefit of "King's."

### THE PRUDENTIAL ASSURANCE COMPANY.

In many ways the "Prudential" is a corporation which stands in a class by itself: it cannot be compared with any other insurance society because there is none which can challenge comparison with it. The scale of its operations is so immense, the influence it exercises so widespread, that shrewd business men were to be found who suggested in the days of the Health Insurance Bill debates that Mr. Lloyd George would have done well to buy up the Prudential for the Government and let that organisation take over the whole administration of the Act. What the directors of the company thought of that idea did not transpire, for the then Chancellor did not adopt the suggestion; but, at least, it is pretty certain that the Act would not be in its present condition of bankruptcy if the scheme had been put through. The recent report of this marvellous company shows that the directors are pursuing their usual prudent policy. In order to maintain the absolute security of the policy-holders the most drastic treatment of the assets and reserves has been adopted. Enormous sums are placed to reserve instead of being paid to shareholders and staff. Both these classes have lost entirely their usual bonus for the year, and the former are docked also of one-fifth of their usual dividend. Policy-holders, too, have to be content with considerably diminished bonus additions; but in view of the circumstances of the war there can be little doubt that the directors are right in their policy of conservation. As a result of their methods the Prudential's name for security will stand higher than ever.

### TROPICAL MEDICINE AND THE ROYAL SOUTHERN HOSPITAL, LIVERPOOL.

In his speech at the recent annual meeting of the Royal Southern Hospital, the President, Mr. Thomas Woodsend, remarked that it was a matter of great pride to report that two members of the nursing staff had been distinguished during service at the Front, Nurse Clark being awarded the Royal Red Cross, and both she and Nurse Casserley being mentioned in dispatches. Three former resident medical officers had also distinguished themselves conspicuously, Dr. Noel Chavasse being mentioned in dispatches and getting the Military Cross, while Dr. Alwyn Smith was awarded the D.S.O.; and Dr. Carey Evans was also mentioned in dispatches. As the beds set aside for wounded or sick soldiers have not been fully occupied during the past twelve months, in view of the waiting list the committee has arranged that twenty of the beds earmarked for soldiers may be used temporarily for civil patients. We note also that the children's out-patient clinic is a development of the children's ward of the hospital proper. Miss Brunton, a lady officially connected with the Invalid Children's Association, assists the staff in the details of this work. The chief event to notice, however, is that the connection between the Royal Southern Hospital and the Liverpool School of Tropical Medicine has been recently severed after a close co-operation during seventeen years, chiefly owing to the school's development under the shadow of the University. But the committee conclude that there is still scope for their tropical diseases ward, if only because of the hospital's close proximity to the docks. Through the severance of the Liverpool School of Tropical Medicine the committee fear to lose the grant of £200 which the school has given to the hospital for many years; and they appeal to the merchants and shipowners of Liverpool who have their business in tropical countries to help the work, which will still continue.

### THE LAST WORD IN FIRE PRECAUTIONS.

One of the things of which the war has taught many hospitals the importance has been the necessity of regular fire-drills and precautions generally. The presence of wounded has also emphasised the need for providing means by which bedridden patients can be quickly removed in case of emergency. It has become customary in many hospitals to tie ribbons to the beds of those patients who are not in a condition to help themselves, and for safety-slings and stretcher-poles to be provided throughout the wards. Where this plan is adopted special fire-drills are necessary to accustom the staff to their use. The possibility of raids from air-craft has also had its effect in making these precautions a reality, which they have not been in the past at many hospitals. The furnishing of appliances on the one hand, and the holding of drills on the other, has been fairly common, but only the possibility of an air-raid, or the chance of its recurrence, has been sufficiently impressive to cause a lively interest in what at ordinary times can hardly escape being regarded as a tiresome routine.

### A PIONEER OF AFTER-CARE.

It is interesting to recall that one of the first persons to realise the importance of after-care was an institutional chaplain, and the work in which he was engaged may claim perhaps to have been one of the latest branches of hospital administration to seek or receive improvement. The chaplain was the late Rev. H. Hawkins, who in 1879, as a result of his experiences as chaplain of the Sussex County Asylum, Haywards Heath and elsewhere, founded the Mental After-care Association. In 1886 active work began, and the late Dr. Hack Tuke was elected first chairman. We recall these facts because they are at once an inspiration to all institutional chaplains by showing what power for good can be exerted by a discerning and enthusiastic man who holds such a post, and also because the Association still hardly receives its due of public recognition. This is apparent from the difficulties which it still has to face, and from the fact that it remains a charity unique of its own kind to-day. In spite of much varied effort, after-care is still a somewhat haphazard affair, and since the problems with which it has to deal embrace the whole talent market, for instance, some greater measure of centralisation would be an improvement. That has yet to come, but when it comes the time will be ripe to remember the pioneer services of the Association that the late Mr. Hawkins founded.

### CONSUMPTIVES IN THE ARMY: A USEFUL SUGGESTION.

The subject of the tuberculous soldier promises to be a topic for discussion both in and out of Parliament for some little time to come. It is well known to tuberculosis officers and sanatorium doctors that numbers of former patients have enlisted in the Army, and it is inevitable that a large proportion of these men should break down under the strain to which they are subjected. The State, therefore, has to incur the cost of equipment and training and is left with a chronic invalid, whom some members of Parliament are anxious to see pensioned for life, although in large numbers of cases there can be no question that the result has not been due to disease "directly and wholly caused by military service." A useful suggestion for eliminating at least some of the cases of soldiers who have been already under treatment for pulmonary tuberculosis before enlistment has been communicated to the editor of *Truth*, and is incorporated in an article in a recent issue. It is that use should be made of the information available in the records of notifications of cases of tuberculosis under the Public Health Regulations, 1912. From these lists the names of men of military age can be obtained, and it should be possible to trace those who have enlisted. In the interests both of the State and of the individual men they should be discharged from the Army.

### PROVISION FOR THE INSANE.

The pressure upon the accommodation at the North Wales Counties Lunatic Asylum, Denbigh, has become so great that it has been proposed to enlarge the asylum at a cost of £40,000. In order to obviate this expenditure a conference of the Boards of Guardians of the five counties interested has suggested that 255 of the harmless asylum inmates should be boarded out amongst four of the Poor-Law institutions. This measure of relief is one which the Lunacy Laws permit. The ordinary workhouse is not, however, well adapted as regards its staff or its equipment to house the insane. The conference would have acted more wisely if it had suggested emptying one of the workhouses and handing it over to the asylum committee to be used as an auxiliary asylum.

### EAST SUSSEX TUBERCULOSIS SCHEME.

The comprehensive county scheme for the treatment of tuberculosis in East Sussex, concerning which negotiations are still proceeding between the County Council and the Insurance Committee, is based on lines now sufficiently familiar owing to the adoption of similar schemes in various parts of the country. In East Sussex the capitation grant to be paid by the Insurance Committee to the

County Council has been reduced from 8d. to 7½d. On behalf of the Council it is recommended that a dispensary be established at Bexhill instead of at Hastings, provided the Insurance Committee agree to there being no dispensary at Hailsham. At the latter place and at Hastings the number of cases is much below the average, while Bexhill is more accessible for the tuberculosis officer. The recommendations of the Insurance Sub-Committee were made with considerable reluctance; this body was loth to hand over to another authority matters for which the Insurance Committee has hitherto been responsible. But the report was eventually agreed to, though the matter was adjourned for a month.

### MARGARINE VERSUS BUTTER.

THE substitution of margarine for butter in the dietary of Poor-Law institutions has been debated recently by many Boards of Guardians with varying results. At Belfast, despite the recommendation of the Local Government Board and the presentation of accounts showing that the change would effect a saving of £1,040 annually, the Guardians decided against the use of margarine. They were no doubt influenced by an adverse report from the visiting medical officers. It would be interesting and valuable to have the reasons for this expression of opinion set out in detail. Those who have had personal experience of the value of margarine cannot help thinking that much of the opposition to it is the result simply of prejudice. The one objection raised at Belfast was that there was no recognised standard for margarine. This is a very real drawback, and we referred to it in THE HOSPITAL of February 19. A short Act embodying a standard to which all articles sold as margarine must attain would be of great practical value.

### CONTRACTS FOR MEDICAL SUPPLIES.

THE purchasing committees of voluntary hospitals and of Poor-Law infirmaries are faced at the present time with great difficulties in obtaining contracts from manufacturers for drugs, dressings, and similar hospital supplies: and there are divergencies of opinion amongst experienced officials who have to advise on such contracts, as to the most advantageous methods to pursue. This question, so far as drugs are concerned, is discussed in an article on another page of the present issue. Few contractors will take contracts for as long as twelve, or even as six months, and contracts for three months hardly signify more than a forward purchase of a definite quantity of goods to be supplied during a given period at a certain price. The institution does, it is true, obtain the advantage of not having to store or pay for a large quantity of goods at once. Whether it would be wise for managers to enter upon contracts for twelve months, assuming that they could find contractors agreeable to it, is also a matter of some doubt. Some urge that when the war ends there will be a sudden drop in prices: others believe that it will be long before markets settle down even if an end of the calamitous struggle were arrived at unexpectedly early.

### NURSES VICTIMS OF FALSE ECONOMY

Is it better for hospital sisters to suffer rheumatism by getting wet for want of protection, or to provide them with a covered way between the hospital wards and their own quarters? After a heated discussion the Hemel Hempstead Joint Isolation Hospital Board decided that it was preferable for the want of protection which exists at present to continue. The proposal of the Mayor, Mr. G. A. Talbot, that estimates be obtained for the provision of a covered way, which, he was advised, would not cost more than £70, was defeated. In spite of the fact that the medical officer, Dr. Burnet, described the covered way as an absolute necessity; in spite of the statements of the matron, Mrs. Chisman, that the nurses got very wet indeed in inclement weather, and though suffering severely from rheumatism, had not made this a cause of complaint, the non-progressive element at the meeting, led by the Chairman, Mr. H. F. Herbert, opposed the provision of a covered way, partly on the ground that it would be extravagant at the present time so to spend the money, and partly because, in his own phrase, the design of the hospital could not be altered to suit any nurses' whims. It will be apparent from this that the tone adopted by the opposition was even more unfortunate than the opposition itself. No suggestion was even made for an alternative provision, and this want of apprehension reflects seriously on Mr. Herbert's aptitude for hospital management. However the hospital was designed, if the original plan proves inadequate for its object, it is only common sense to adopt a remedy.

### THIS WEEK'S DRUG MARKET.

BUSINESS continues good, but while prices are, on the whole, well maintained, few substantial advances have taken place. Cinchona bark is much dearer, but quinine is quiet, and there is no noteworthy alteration in price. Potassium permanganate is again dearer, and quotations for borax and boric acid have been raised. Menthol is again dearer, and belladonna root is scarcer than ever. Acetanilide and phenacetin have a slightly improved tendency, but hexamine is again rather lower. The demand for bromides continues quiet, and although it might be possible to buy at slightly below makers' quotations, no very large quantities could be procured at anything under those figures at present. Quotations for cod-liver oil differ considerably, but business is actually reported at prices which in ordinary times would be regarded as fabulous; the effect of these high figures will undoubtedly be to curtail the demand very seriously, and it would probably be unwise to purchase at present more cod-liver oil than is necessary to cover immediate requirements. Of gum tragacanth there appears to be very little good quality available, and any small lots offered would fetch very high rates. The value of cocaine is firmly maintained. but where there is no immediate need to replenish stocks buyers would perhaps be wise to delay making purchases for the present.

## Figures and Tables

**Figure f1:**